# Unbound free fatty acids from intralipid displace bilirubin from albumin, comparable to sulfisoxazole

**DOI:** 10.1038/s41390-025-04673-y

**Published:** 2025-12-02

**Authors:** Thomas Hegyi, Andrew Huber, William Oh, Alan Kleinfeld

**Affiliations:** 1 The Division of Neonatologéy, Department of Pediatrics, Robert Wood Johnson Medical School, Rutgers University, New Brunswick, NJ USA; 2 Fluoresprobe Sciences and Torrey Pines Institute for Molecular Studies, San Diego, CA USA; 3 Department of Pediatrics, Warren Alpert Medical School of Brown University, Providence, RI USA

## Abstract

**Background:**

Kernicterus can occur even with seemingly safe total bilirubin levels if the unbound bilirubin fraction (Bf) increases. This elevation can be caused by substances that displace bilirubin from albumin. Sulfisoxazole is a known displacer, and our previous research suggested that Intralipid might have a similar effect.

**Methods:**

Our study aimed to compare the bilirubin-displacing effects of unbound free fatty acids (FFAu) from Intralipid with those of sulfisoxazole. We used a modified bilirubin fluorescence sensor to measure Bf in undiluted samples. Bilirubin–albumin complexes were created using human serum albumin (3 g/dL) and bilirubin (257 µmol/L), and FFAu was quantified with the ADIFAB2 sensor. Displacement studies involved titration with either sulfisoxazole or FFAu components of Intralipid, including oleate and linoleate.

**Results:**

The baseline Bf was 0.017 µmol/L. Sulfisoxazole at 540 µmol/L raised Bf to 0.070 µmol/L. Comparable increases were observed with unbound oleate and linoleate at approximately 0.200 µmol/L and 1.800 µmol/L, respectively.

**Conclusions:**

FFAu from Intralipid displaces bilirubin from albumin as effectively as sulfisoxazole at concentrations associated with kernicterus. These findings emphasize the potential neurotoxicity risk of Intralipid use in vulnerable infants and stress the importance of monitoring Bf and reassessing lipid therapy protocols in the NICU.

**Impact:**

- Unbound free fatty’ acids (FFAu) from Intralipid displace bilirubin from albumin as potently as sulfisoxazole, a known bilirubin-displacing drug, thereby elevating unbound bilirubin (Bf) to levels associated with kernicterus. This identifies Intralipid as a potential contributor to bilirubin neurotoxicity risk in preterm and sick newborns.
- Addition to literature: Provides direct quantitative evidence, using a bilirubin fluorescence sensor, that FFAu components of Intralipid (especially oleate and linoleate) displace bilirubin from albumin and extends prior suggestions and in vitro observations by showing equivalence to sulfisoxazole, the classic displacer historically linked to kernicterus.
- Impact: Raises clinical concern about the safety of Intralipid administration in vulnerable neonates, particularly preterm infants with impaired bilirubin clearance and reduced albumin binding capacity and suggests that lipid therapy protocols in the NICU may inadvertently increase kernicterus risk, calling for careful evaluation of dosing, timing, and monitoring.

## Introduction

Neonatal hyperbilirubinemia is common and usually harmless, but in certain situations, it can cause severe neurotoxicity and kernicterus. In the past, the risk of bilirubin-induced neurological dysfunction (BIND) has been associated with high total serum bilirubin (TSB) levels.^1,2^ However, unbound bilirubin (Bf), which is the free, non-albumin-bound portion, is now considered a more accurate indicator of neurotoxicity because it can cross the immature blood-brain barrier and damage neuronal tissue.^3,4^

Several agents, including sulfonamides like sulfisoxazole, have been linked to kernicterus due to their ability to displace bilirubin from albumin binding sites.^5–7^ In the neonatal intensive care unit, lipid emulsions such as Intralipid are commonly administered to preterm infants to meet their caloric and essential fatty acid needs. However, growing evidence indicates that unbound free fatty acids (FFAu) produced from Intralipid metabolism may competitively displace bilirubin from albumin.^8,9^

We hypothesized that the bilirubin-displacing effect of FFAu derived from Intralipid is comparable in magnitude to that of sulfisoxazole, a known kernicterogenic agent. The study aimed to directly compare the displacement capacity of sulfisoxazole with oleate and linoleate, the main FFA components in Intralipid, using a new sensor-based method to measure Bf in undiluted samples.

## Methods

Human serum albumin (HSA, Sigma-Aldrich) was reconstituted to a final concentration of 3 g/dL. Bilirubin (Sigma-Aldrich) was dissolved in dimethyl sulfoxide (DMSO) and added to achieve a final concentration of 257 µmol/L, resulting in a molar bilirubin-to-albumin ratio of approximately 0.5, which simulates physiological conditions in extremely low birth weight (ELBW) infants.

We used a new Bf sensor with near-infrared (NIR) fluorophores (LI-COR Biosciences) to measure Bf at 22 °C in undiluted samples. NIR fluorescence is mostly unaffected by bilirubin, hemoglobin, or other blood components, enabling accurate Bf measurements in both serum and whole blood.^10^

FFAu levels were measured using the ADIFAB2 sensor (FFA Sciences), which provides real-time fluorescent detection of unbound fatty acids in biological media.^11^ Titration experiments involved increasing concentrations of sodium oleate and linoleate (Cayman Chemical), mimicking the primary FFA components in Intralipid emulsions.

Aliquots of bilirubin-HSA complexes were titrated with increasing concentrations of sulfisoxazole (Sigma-Aldrich), sodium oleate, or linoleate. Sulfisoxazole was added up to a maximum concentration of 900 µmol/L, exceeding the 540 µmol/L threshold previously associated with kernicterus in clinical reports.^7,12^ FFAu concentrations were increased to simulate the peak FFAu values seen in neonates receiving parenteral lipid nutrition.^8,13^

## Results

The assessment of sensor performance and assay validation showed that the NIR-based Bf sensor accurately detected Bf in whole blood, with no interference from bilirubin or hemoglobin.^14^ Assay precision was high, with intra-assay variability under 10% and minimal background fluorescence. The FFAu measurements from the ADIFAB2 sensor also showed consistent linear responses to oleate and linoleate additions in the nanomolar range.

At baseline, the prepared bilirubin–albumin complexes (HSA 3 g/dL, bilirubin 257 µmol/L; molar ratio 0.5) demonstrated a low unbound bilirubin (Bf) concentration of 0.017 ± 0.002 µmol/L, consistent with strong bilirubin binding to albumin under physiological conditions.

**Fig. 1:**
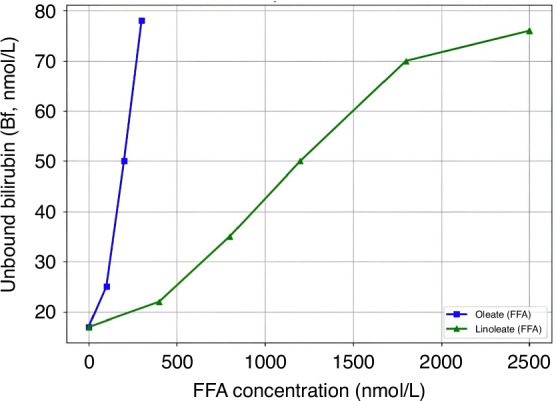
Effect of free fatty acids on unbound bilirubin. Increasing concentrations of Oleate and Linoleate are associated with increasing levels of unbound bilirubin.

Table 1 shows the Bf levels at increasing sulfisoxazole concentrations and Fig. 1 shows the dose-response curves of Bf versus displacer concentration for oleate and and linoleate. Titration with sulfisoxazole caused a dose-dependent increase in Bf, rising from 0.030 ± 0.003 µmol/L at 100 µmol/L to 0.08 ± 0.006 µmol/L at 9 µmol/L. At the clinically relevant concentration of 540 µmol/L, which has been linked to kernicterus in neonates,^3^ Bf reached 0.07 ± 0.005 µmol/L. The smaller increase in Bf from 540 to 940 µmol/L of sulfisoxazole, compared to the rise from 100 to 540 µmol/L, indicates saturation of displacement capacity at higher concentrations. These findings confirm previous observations that sulfisoxazole is a potent displacer of bilirubin from albumin.

**Table 1:** Unbound Bilirubin (Bf) at Increasing Sulfisoxazole Concentrations

Sulfisoxazole (µmol/L)	Bf (µmol/L)
0	0.030
5	0.050
10	0.070
20	0.075
30	0.080

Titration with sodium oleate resulted in a similarly steep rise in Bf, reaching 0.070 ± 0.005 µmol/Lat approximately 0.200 µmol/L FFAu. This level matched that induced by 540 µmol/L sulfisoxazole. A plateau was observed at 0.300 µmol/L, where Bf peaked at 0.078 ± 0.006 µmol/L. Linoleate exhibited similar, though less pronounced, displacement effects and reached the same displacement threshold of 0.070 ± 0.005 µmol/L of Bf at approximately 1.800 µmol/L FFAu.

Comparing potency and displacement efficiency at equivalent Bf levels ( ~ 0.070 µmol/L), sulfisoxazole required 540 µmol/L, oleate required approximately 0.200 µmol/L, and linoleate required approximately 1.800 µmol/L. This results in a displacement potency ratio of roughly: Sulfisoxazole : Oleate : Linoleate = 1 : 0.0004 : 0.0033 (on a molar basis). These differences emphasize oleate’s high displacement efficiency, even at nanomolar concentrations typically reached during Intralipid infusion in newborns.^14,15^ These data indicate that linoleate, although requiring higher concentrations than oleate, still attains Bf levels associated with bilirubin neurotoxicity risk.

## Discussion

This study shows that free fatty acids released from Intralipid metabolism can displace bilirubin from albumin as effectively as sulfisoxazole, an antibiotic known to be associated with kernicterus. This highlights a critical and often overlooked risk in neonatal care. Although sulfisoxazole has been removed from neonatal use because of its well-documented link to kernicterus,^16^ Intralipid remains essential in providing nutritional support to premature infants. The current study finds that FFAu components of Intralipid, especially oleate and linoleate, can increase Bf to levels similar to those caused by sulfisoxazole, raising important safety concerns.

Albumin’s high-affinity binding of bilirubin primarily takes place at Sudlow site I, which is susceptible to competitive or allosteric inhibition by various ligands, including fatty acids and drugs.^17–19^ Sulfisoxazole is known to displace bilirubin by binding at or near this site.^5,20^ Similarly, long-chain polyunsaturated fatty acids, such as oleate and linoleate, also bind to albumin with enough affinity to displace bilirubin.^21^ Notably, in this study, oleate displaced bilirubin to similar Bf levels at concentrations that are an order of magnitude lower than linoleate, indicating higher binding potency and/or greater occupancy of the albumin site, consistent with previous structural and spectroscopic analyses.^22^

These findings have important implications for NICU practice. Increasing evidence suggests that Bf, not TSB, should be the primary marker used to assess bilirubin neurotoxicity risk.^17,23^ Bf levels as low as 0.070 µmol/L have been associated with adverse auditory and neurological outcomes in premature infants.^24,25^ The findings presented here are especially concerning given the vulnerability of extremely low birth weight (ELBW) infants, who often have lower circulating albumin levels, immature hepatic glucuronidation, increased blood-brain barrier permeability,^26^ and greater exposure to stressors (e.g., hypoxia, acidosis, sepsis) that further raise Bf levels.^27^ These issues have recently gained attention in clinical practice, showing that free fatty acids from Intralipid administration can separate Bf from TSB, potentially weakening the effectiveness of phototherapy, and that higher doses of Intralipid increase Bf concentration in preterm infants.^8,28^

Our findings challenge the assumption that lipid emulsions are harmless, especially in infants with immature albumin-binding capacity, acidosis, or competing binding substances. Therefore, even with normal or low TSB levels, these infants may still reach neurotoxic thresholds due to increased displacement of bilirubin from albumin by FFAu.^29^ Based on our findings, we recommend the following changes to clinical practice to protect infants from the hidden risks associated with elevated Bf levels: 1) Routinely monitor Bf in at-risk neonates, particularly ELBW infants receiving lipid infusions, 2) Reassess lipid dosing protocols to consider lower initial doses and slower titration, especially during the first 72 hours of life, 3) Explore safer lipid emulsions with reduced oleate and linoleate content or those with structured triglycerides that release less FFAu during metabolism,^15^ and 4) Incorporate Bf data into clinical decision-making algorithms alongside gestational age, albumin levels, and acid–base status.

## Conclusion

Our data demonstrate that unbound free fatty acids from Intralipid displace bilirubin from albumin with an effectiveness similar to that of sulfisoxazole, a drug historically associated with kernicterus. The effects of Intralipid on Bf displacement are dose-related, with maximal effect at dosages equal to or greater than 3 g/kg/day.^28^ These findings highlight a potential iatrogenic factor contributing to bilirubin neurotoxicity in preterm infants and suggest that lipid administration practices in the NICU should be reassessed, with a focus on Bf monitoring^29^ and reducing FFAu exposure. Future research should include: (1) longitudinal cohort studies linking FFAu, Bf, and neurodevelopmental outcomes; (2) biophysical binding models to measure the displacement constants of different FFA species, which could lead to safer lipid formulations; and (3) NICU trials comparing Bf-targeted lipid management with traditional TSB-driven protocols to lower the risk of BIND.^30^

## Data Availability

The datasets generated and/or analyzed during the current study are available from the corresponding author upon reasonable request.
